# Macrophage-derived Lipocalin-2 contributes to ischemic resistance mechanisms by protecting from renal injury

**DOI:** 10.1038/srep21950

**Published:** 2016-02-25

**Authors:** Michaela Jung, Bernhard Brüne, Georgina Hotter, Anna Sola

**Affiliations:** 1 Institute of Biochemistry I, Goethe-University Frankfurt, Theodor-Stern-Kai 7, Frankfurt am Main, Germany; 2 Department of Ischemia and Inflammation, IIBB-CSIC-IDIBAPS, Barcelona, Spain; 3 CIBER-BBN, Networking Centre on Bioengineering, Biomaterials and Nanomedicine (CIBER-BBN), Barcelona, Spain; 4 Department of Experimental Nephrology, IDIBELL, L’Hospitalet del Llobregat, Barcelona, Spain

## Abstract

Renal ischemia-reperfusion injury triggers an inflammatory response associated to infiltrating macrophages which determines the further outcome of disease. Brown Norway rats are known to show endogenous resistance to ischemia-induced renal damage. By contrast, Sprague Dawley rats exhibit a higher susceptibility to ischemic injury. In order to ascertain cytoprotective mechanisms, we focused on the implication of lipocalin-2 protein in main resistance mechanisms in renal ischemia/reperfusion injury by using adoptive macrophage administration, genetically modified *ex vivo* either to overexpress or to knockdown lipocalin-2. *In vitro* experiments with bone marrow-derived macrophages both from Brown Norway rats and from Sprague Dawley rats under hypoxic conditions showed endogenous differences regarding cytokine and lipocalin-2 expression profile in the two strains. Most interestingly, we observed that macrophages of the resistant strain express significantly more lipocalin-2. *In vivo* studies showed that tubular epithelial cell apoptosis and renal injury significantly increased and reparative markers decreased in Brown Norway rats after injection of lipocalin-2-knockdown macrophages, while the administration of lipocalin-2-overexpressing cells significantly decreased Sprague Dawley susceptibility. These data point to a crucial role of macrophage-derived lipocalin-2 in endogenous cytoprotective mechanisms. We conclude that expression of lipocalin-2 in tissue-infiltrating macrophages is pivotal for kidney-intrinsic cytoprotective pathways during ischemia reperfusion injury.

Acute renal failure, most commonly induced by ischemia/reperfusion injury (IRI) affects about 5% of all hospitalized patients. Despite recent advances in preventive strategies, it still counts with significant morbidity and a high rate of mortality. Most of the published studies focus on mechanisms of injury induction and the following events during the development of IRI^1^,^2^,^3^, but little is known about the endogenous potential of the kidney to recover from IRI. Intrinsic resistance to injury and the correlated mechanisms would provide a good starting point for therapeutic approaches. Elucidation of relevant mechanisms and/or mediators would facilitate the protection of the kidney from injury and to limit the pathogenesis of acute renal failure.

Galinanes *et al.*^4^ suggested a genetic origin for endogenous resistance against myocardial protection in response to IRI, based on differences between species in ventricular pressure, creatinine kinase leakage, and tissue high-energy phosphates. In this line, and concerning renal IRI, it seems that the kidney follows the same pattern as seen for different levels of resistance to injury between the two species, Sprague Dawley and Brown Norway rats^5^. Evidences point out that resistance of Brown Norway rats to ischemia is determined by a favourable balance of oxidant production versus oxidant removal, indicated by decreased apoptosis, necrosis and different resolution of the inflammation^6^,^7^,^8^.

The intrinsic response to ischemic acute renal failure involves a complex cascade of mechanisms. In particular, macrophage infiltration is a crucial feature associated to the severity of renal injury and the progressive renal failure. However, it is also a determinant event that facilitates regeneration and repair, for both cases depending on the microenvironment and on their activation status. During the acute phase of tissue injury, macrophages show a clear pro-inflammatory phenotype and infiltration intensity correlates with injury severity and contributes to tissue destruction. Previous studies from our group delineated a pivotal role for the macrophage phenotype influencing the inflammatory environment and determining kidney repair^9^,^10^,^11^. Furthermore, other studies also confirmed a beneficial role for macrophages depending on their phenotype and nature of injury^12^.

Lipocalin-2 (Lcn-2), also called Neutrophil gelatinase-associated lipocalin (NGAL) has been described as a marker and potential positive modulator of acute inflammation. Lcn-2 is a 25 kDa secreted protein of the lipocalin superfamily of carrier proteins^13^ that is known to be synthesized from macrophages^14^ and to have the ability to induce cell death in specific immune cell populations. Interestingly, non-hematopoietic cells and monocyte-derived macrophages were resistant, whereas neutrophils and primary lymphocytes underwent apoptosis^15^. Lcn-2 is also expressed in kidney cells, and its production markedly increases in response to stimuli such as ischemia or nephrotoxic agents. Hence, Lcn-2 has been assigned a dual role both as a proapoptotic, but also as a prosurvival factor inducing proliferation and ameliorating tubule cell apoptosis^16^,^17^,^18^. Data from our group suggests that endogenously generated Lcn-2-induced renal cell regeneration depends on inflammatory cytokines in mouse kidney IRI^9^,^19^. Taken into account the explained above, Lcn-2 may be one of the potential modulators playing a key role in macrophage phenotype determination and resolution of inflammation, thus promoting renal epithelial cell regeneration and defining injury outcome.

In view of the above, we hypothesized that endogenous resistance to ischemic injury could be determined by the nature of the macrophage phenotype. More concretely, we point out that the expression of Lcn-2 from infiltrated macrophages could be determinant to define IRI sensibility. Thus, the focus of this study was the design of a cellular therapy approach based on the utilization of pro-reparative macrophages by overexpression of Lcn-2 in order to foster kidney repair after IRI in IRI-sensitive Sprague-Dawley rats. Furthermore, by means of adoptive Lcn-2-knockdown macrophage administration, we could show a direct implication of macrophage Lcn-2 in main cytoprotective mechanisms in IRI-resistant Brown Norway rats.

## Results

### Bone marrow-derived macrophages from Brown Norway and Sprague Dawley rats show a different response to hypoxia/re-oxygenation *in vitro*

**Figure 1:**
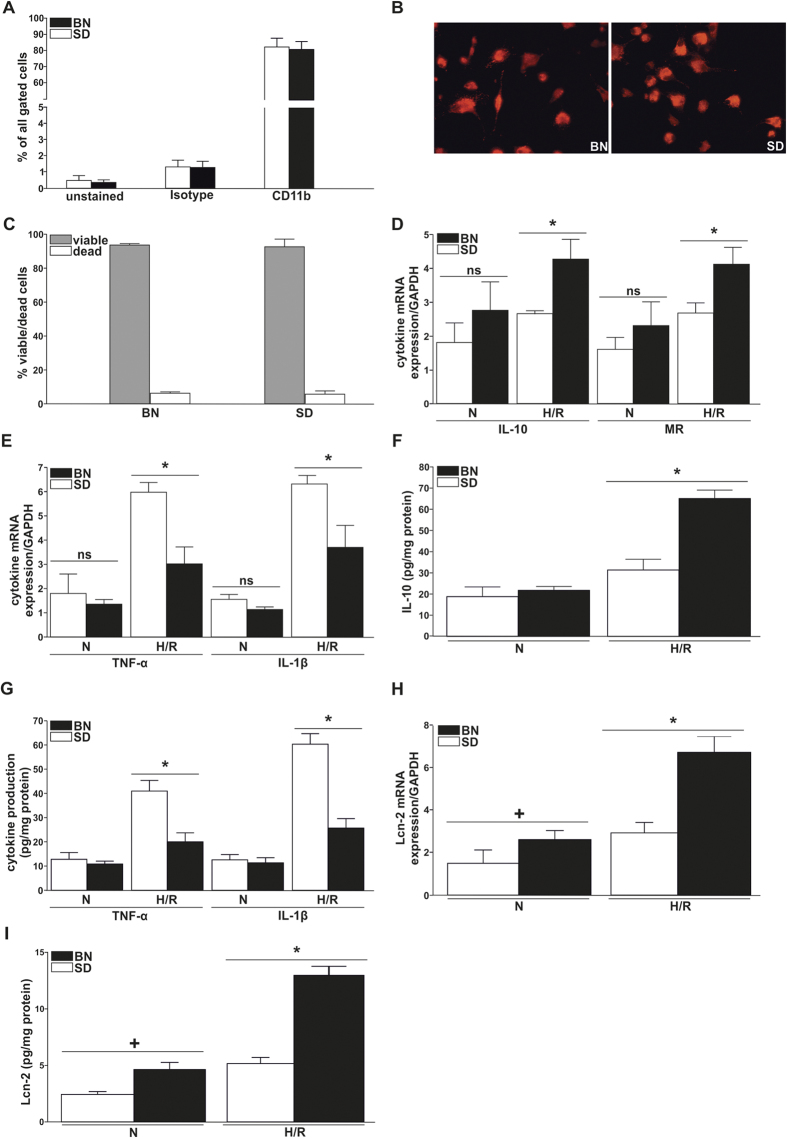
Bone marrow-derived macrophages (BMDM) from Brown Norway and Sprague Dawley rats respond differently to hypoxia/re-oxygenation *in vitro.* (**A**) The assessment of CD11b levels by flow cytometry demonstrates clear maturity and differentiation of precursor cells from the bone-marrow towards macrophages after incubation with GM-CSF (10 ng/ml) for 7 days. (**B**) Immunofluorescent staining of ED1 (CD68) of BMDM showed high numbers of ED1 positive cells out of the total number of cells differentiated from whole bone marrow preparations, thus confirming the maturity status of differentiated macrophages. A representative cross section of at least 5 independent experiments is illustrated. (**C**) FACS analysis of propidium Iodid (PI) and annexin V binding of differentiated BMDM from both strains shows no significant differences in viability. **(D–I)** BMDM from SD and BN rats were either exposed to hypoxia (1% oxygen) for 4 h and then re-oxygenated (20.7% oxygen) for 16 h or left under normoxic (20.7% oxygen) conditions. (**D**) Anti-inflammatory (IL-10, MR) and (**E**) pro-inflammatory (TNF-α, IL-1β) cytokine expression was quantified on mRNA level using qRT-PCR. Results show no significant differences under normoxic conditions (N), whereas a significantly higher anti-inflammatory cytokine expression can be observed for BN macrophages after hypoxia/re-oxygenation (H/R) as compared to H/R-group from SD macrophages. These results were further corroborated by measuring protein levels for (**F**) IL-10, as well as (**G**) TNF-α and IL-1β by ELISA. Lcn-2 expression was measured on (**H**) mRNA by real-time qRT-PCR and on (**I**) protein level by ELISA. Results show significantly elevated levels of Lcn-2 expression in BN macrophages as compared to SD, both under normoxic and H/R conditions. qRT-PCR data was normalized to relative expression of the housekeeping gene GAPDH and was presented as arbitrary units of relative expression. Data are represented as means ± S.E.M; n = 8, *p < 0.05 BN H/R vs. SD H/R, ^+^ p < 0.05 BN N vs. SD N.

The maturity status of bone marrow-derived macrophages (BMDM) isolated from both rat strains showed no differences in their ability to differentiate to macrophages from monocytic precursors. These results were obtained by flow cytometry analyzing CD11b on the surface of BMDMs, which is preferentially expressed in myeloid cells and widely used as a marker for macrophage maturity. Approximately 80–85% (CD11b positive cells in %: Isotype BN 1.41+/−0.35, SD 1.29+/−0.38; CD11b BN 81.91+/−4.51, SD 80.88+/−4.95) of the bone marrow cells differentiated into macrophages after 7 days of GM-CSF-induced (10 ng/ml, applied daily) maturation (Fig. 1A). Immunostaining for ED1, another commonly accepted macrophage marker (Fig. 1B), corroborated flow cytometry results and showed a similar maturity status for BMDMs of both strains, showing nearly 100% ED1-positive cells of total BMDMs. Trypan Blue exclusion assays indicated that BMDMs from both strains were about 95% viable (Fig. 1C).

Next, we applied an *in vitro* system of hypoxia/re-oxygenation (H/R) that mimicked the *in vivo* situation of IRI. For this purpose, BMDMs from each strain were exposed to hypoxia (1% oxygen) for 4 h, and then re-oxygenated (20.7% oxygen) for 16 h. We chose these time-points according to supplementary table 1 in order to balance the amount of hypoxia-induced molecular changes and downstream effects on cellular viability. We then analyzed the expression of the anti-inflammatory mediators IL-10 and Mannose Receptor (MR), as well as the pro-inflammatory cytokines IL-1β and TNF-α at mRNA (Fig. 1D,E) and protein (Fig. 1F,G) level. BMDMs from Brown Norway (BN) rats expressed significantly higher levels of anti-inflammatory mediators in response to H/R than their counterparts isolated from Sprague Dawley (SD) rats. In contrast, BN-macrophages expressed significantly fewer pro-inflammatory cytokines upon H/R stimulation. Instead, SD-macrophages showed a profound inflammatory response after H/R treatment. Additionally, we were interested in Lcn-2 expression in BMDMs from both strains. BN-macrophages showed significantly higher Lcn-2 expression at both mRNA (Fig. 1H) and protein (Fig. 1I) level than SD-macrophages. This was evident both at baseline and under control conditions, but highly pronounced after H/R.

### Brown Norway and Sprague Dawley rats respond differently to ischemia/reperfusion injury *in vivo*

**Figure 2:**
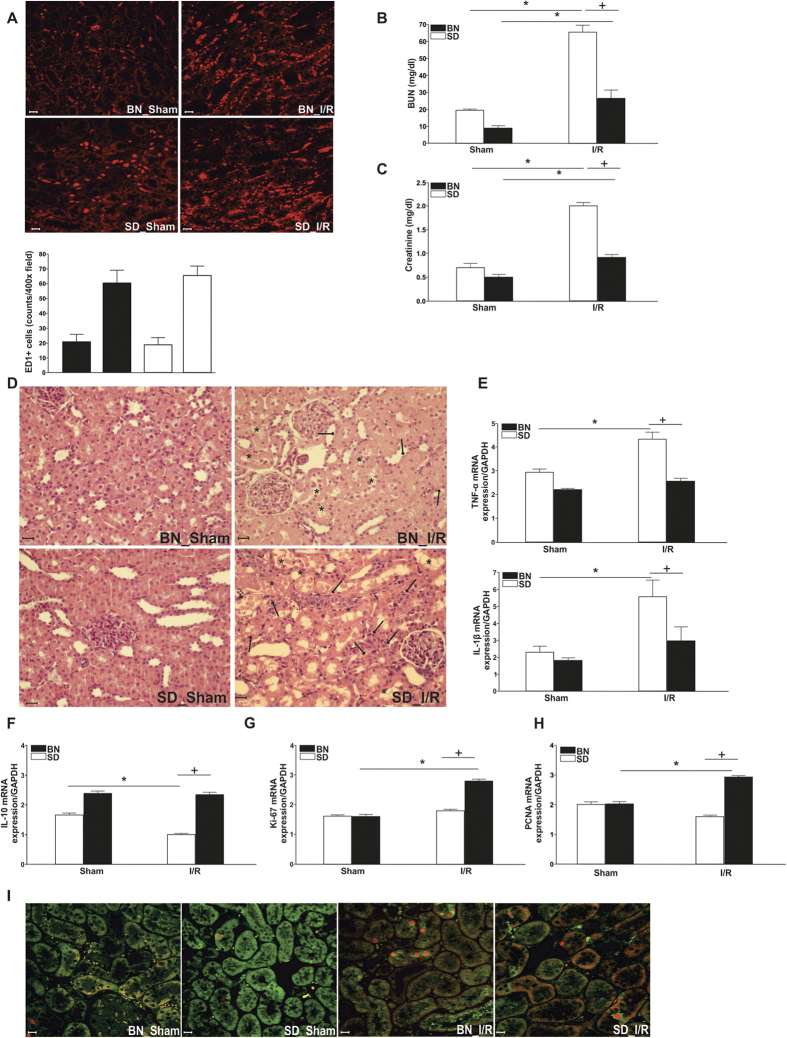
Brown Norway and Sprague Dawley rats respond differently to ischemia/reperfusion injury *in vivo.* Animals were subjected to 45 min. of bilateral ischemia or were sham-operated and sacrificed at 24 h of reperfusion. (**A**) ED1 (CD68) staining of kidney sections from both strains shows comparable numbers of infiltrated macrophages in the tubular interstitium after 24 h of reperfusion. Macrophages were quantified by counting the number of positive cells per 100 cells counted in an average of five high-power fields (x40) in each section. (**B**) Blood urea nitrogen (BUN) and (**C**) creatinine in plasma of both strains was assessed in Sham and I/R group. (**D**) Conventional histological analysis of H&E-stained kidney sections (original magnification x40) of the cortico-medullar region confirmed BUN and creatinine data for both strains. Representative images of Sham rats are shown and compared with I/R groups for each strain. Arrows indicate epithelial cell balloonization and tubular dilatation and asterisks show epithelial necrosis, detachment, and oedema (**E**) TNF-α and IL-1β kidney expression profiles as representative inflammatory milieu and (**F**) IL-10 expression profile as representative anti-inflammatory mediator of the different study groups was measured by mRNA expression via qRT-PCR. Comparing I/R groups of BN and SD animals, SD rats show a significant increase in whole kidney inflammation measured by increased levels of pro-inflammatory markers and a decrease of anti-inflammatory cytokine as compared to BN animals in the I/R group. The effect of (**G**) Ki-67 and (**H**) PCNA mRNA expression (tested via qRT-PCR in total renal tissue homogenates) and (**I**) immunostaining of stathmin (green) and PCNA (red) expression (original magnification x400), in renal tissue of Sham- and I/R-treated groups of both rat strains was assessed. Results show a clear increase of regenerative/proliferative parameters in the BN I/R-group as compared to the SD I/R group. Real-Time qRT-PCR data was normalized to the housekeeping gene GAPDH and expressed as arbitrary units of relative expression. Data is represented as means ± S.E.M. n = 5; *p < 0.05 vs SD/BN Sham respectively, ^+^ p < 0.05 vs. BN I/R. bar = 50 μm.

In order to characterize strain-specific response to ischemia/reperfusion injury, we first checked macrophage infiltration after IRI (Fig. 2A). Representative images of fluorescent stainings for ED1 in either Sham-operated or IRI-treated animals show that substantial macrophage infiltration is not altered between the two rat species. Additionally, we observed a significant increase in macrophage homing to the kidney after 24 h of reperfusion. Quantification of ED1-positive macrophage numbers in renal tissue by counting at least five different fields showed a similar macrophage infiltration index for both strains, being significantly increased after 24 h of reperfusion (Fig. 2A).

Blood urea nitrogen (BUN) (Fig. 2B) and creatinine (Fig. 2C) showed a significant increase at 24 h of reperfusion for both strains used in this study. However, we observed significantly higher levels of both renal functional markers in SD rats as compared to BN rats. H&E staining (Fig. 2D) and histological analysis (Table 1) further corroborated these results. Arrows indicate epithelial cell balloonization and tubular dilatation and asterisks show epithelial necrosis, detachment, and oedema. Furthermore, determination of inflammatory mediators in whole kidney homogenates showed that the pro-inflammatory cytokines TNF-α and IL-1β were significantly induced at mRNA level after 24 h of reperfusion only in SD-rats (Fig. 2E), whereas the anti-inflammatory mediator IL-10 was significantly decreased at 24 h of reperfusion (Fig. 2F). Furthermore, a significant difference in inflammatory tissue outcome could be observed for the different strains at 24 h of reperfusion. SD rats developed a more severe inflammatory environment than BN rats at 24 h of reperfusion, shown by increased inflammatory cytokines and reduced anti-inflammatory mediators. Additionally, we performed immunostaining of Mannose receptor (CD206) (Figure S3A) as a representative M2-macrophage marker and iNOS (Figure S3B) as a representative M1-marker in kidney sections of BN and SD rats. Results showed a significantly increased number of CD206-positive cells in tissues of BN rats, whereas in SD rats the iNOS positive cells are predominant. These findings further corroborate the intrinsic differences in renal macrophages of both strains and underline our previous results.

Next, we determined the expression of the regeneration markers Ki-67 and PCNA at mRNA level in whole tissue homogenate (Fig. 2G,H) and by immunofluorescent staining (Fig. 2I). We observed a significant increase in both repair markers 24 h after ischemia as compared to Sham-operated control animals only for BN rats, and not for SD rats. In line, we showed a significant increase in Ki-67 and PCNA expression in BN rats as compared to SD rats at 24 h of reperfusion. Quantification of PCNA- and stathmin-positive cells by counting at least five different fields further corroborated these results (Table 2).

### Lcn-2 over-expressing macrophages protect against IRI in Sprague Dawley rats

**Figure 3:**
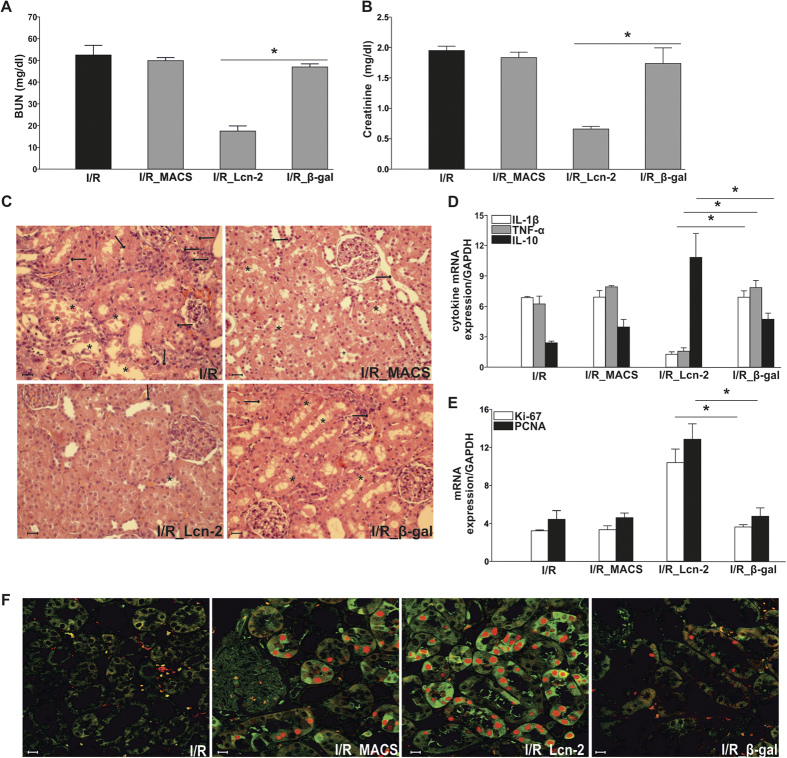
Lcn-2 over-expressing macrophages protect against IRI in Sprague Dawley rats. Animals were subjected to 45 min. of bilateral ischemia or were sham-operated and sacrificed at 24 h of reperfusion. The effect on (**A**) blood urea nitrogen (BUN) and (**B**) creatinine in plasma of adoptive transfer of either β-gal- or Lcn-2-treated macrophages was assessed. Macrophage infusion was performed for each condition at 1 h of reperfusion. The following groups were analyzed: Sham: control group without infusion of macrophages; I/R: bilateral ischemia of 45 min. with subsequent reperfusion of 24 h; I/R_MACS: ischemia/reperfusion group with injection of untreated BMDM; I/R_Lcn-2: ischemia/reperfusion group with adoptive transfer of *ex vivo*-modified macrophages overexpressing Lcn-2; I/R_β-gal: ischemia/reperfusion group administered control β-gal expressing macrophages. (**C**) Conventional histological analysis of H&E-stained kidney sections (original magnification x40) of the cortico-medullar region confirmed BUN and creatinine data for the protective effect of Lcn-2 overexpressing macrophage infusion. Representative images of are shown and compared with I/R_β-gal group. Arrows indicate epithelial cell balloonization and tubular dilatation and asterisks show epithelial necrosis, detachment, and oedema (**D**) TNF-α, IL-1β, and IL-10 kidney expression profiles as representative inflammatory milieu of the different study groups was measured by mRNA expression via qRT-PCR from whole tissue homogenates. Results show the down-regulation of pro-inflammatory cytokines via the adoptive transfer of Lcn-2 over-expressing macrophages as compared to β-gal expressing macrophage infusion. The effect of (**E**) Ki-67 and PCNA mRNA expression (tested via qRT-PCR in total renal tissue homogenates) and (**F**) immunostaining of stathmin (green) and PCNA (red) expression (original magnification x400), in renal tissue of adoptive transfer of either β-gal- or Lcn-2-treated macrophages was assessed. Results show a clear increase of regenerative/proliferative parameters in the Lcn-2 overexpressing group compared to the β-gal macrophage group. qRT-PCR data was normalized to the housekeeping gene GAPDH and expressed as arbitrary units of relative expression. Data is represented as means ± S.E.M. n = 5; *p < 0.05 vs IR_β-gal. bar = 50 μm.

Administration of untreated control macrophages was unable to prevent renal injury measured by BUN (Fig. 3A) and serum creatinine (Fig. 3B) at 24 h of reperfusion. In contrast, adoptively transferred Lcn-2-over-expressing macrophages significantly reduced both BUN and creatinine levels. The administration of control β-gal-adenovirus transduced macrophages showed no amelioration as compared to the I/R group, and both BUN and creatinine levels were comparable to the group with non-transduced control macrophages. Histological analysis (Fig. 3C) and injury quantification (Table 1) indicated that renal injury could be observed at 24 h of reperfusion. Treatment with Lcn-2-macrophages showed significantly higher tissue integrity, whereas transfer of either non-transduced or β-gal-adenovirus transduced control macrophages did not improve injury outcome. Transduction efficiency was evaluated via the transduction with a GFP-tagged AdLcn-2 adenoviral vector and verified by fluorescent microscopy (Figure S1A). AdLcn-2 transduction shows a dose- and time-dependent increase in Lcn-2 expression, both at mRNA (Figure S1B) and protein (Figure S1C) level. We also measured IL-10, Mannose Receptor (MR), TNF-α, and IL-1β production in transduced macrophages. Results show an up-regulation of anti-inflammatory mediators in Lcn-2-macrophages, even when stimulated with the pro-inflammatory mediator LPS (Figure S1D–S1I). This may also occur due to autocrine effects of Lcn-2, since we have indications from cytokine measurements that Lcn-2 may polarize macrophages towards an anti-inflammatory M2-like phenotype. Upon AdLcn-2 transduction, macrophages express higher amounts of anti-inflammatory mediators (Figure S1D, S1E, and S1H) and reduced levels of pro-inflammatory cytokines (Figure S1F, S1G, and S1I), an effect that is even more pronounced under LPS-stimulated conditions *in vitro*.

In line, renal TNF-α and IL-1β expression were significantly reduced in whole tissue homogenates upon administration of Lcn-2-macrophages, whereas the anti-inflammatory mediator IL-10 was significantly increased (Fig. 3D).

The regenerative parameters Ki-67 and PCNA (Fig. 3E) showed a clear increase at mRNA expression in the Lcn-2-overexpressing macrophage-treated groups. Additionally, we performed staining for PCNA and stathmin as commonly used regenerative markers (Fig. 3F). Few cells were found to express stathmin and PCNA in I/R group. Treatment with non-transduced or β-gal-transduced control macrophages showed similar results, whereas Lcn-2-overexpressing macrophages increased their expression. Quantification of PCNA- and stathmin-positive cells by counting at least five different fields further corroborated these results (Table 2).

### Lcn-2 knockdown in macrophages promoted IRI in Brown Norway rats

**Figure 4:**
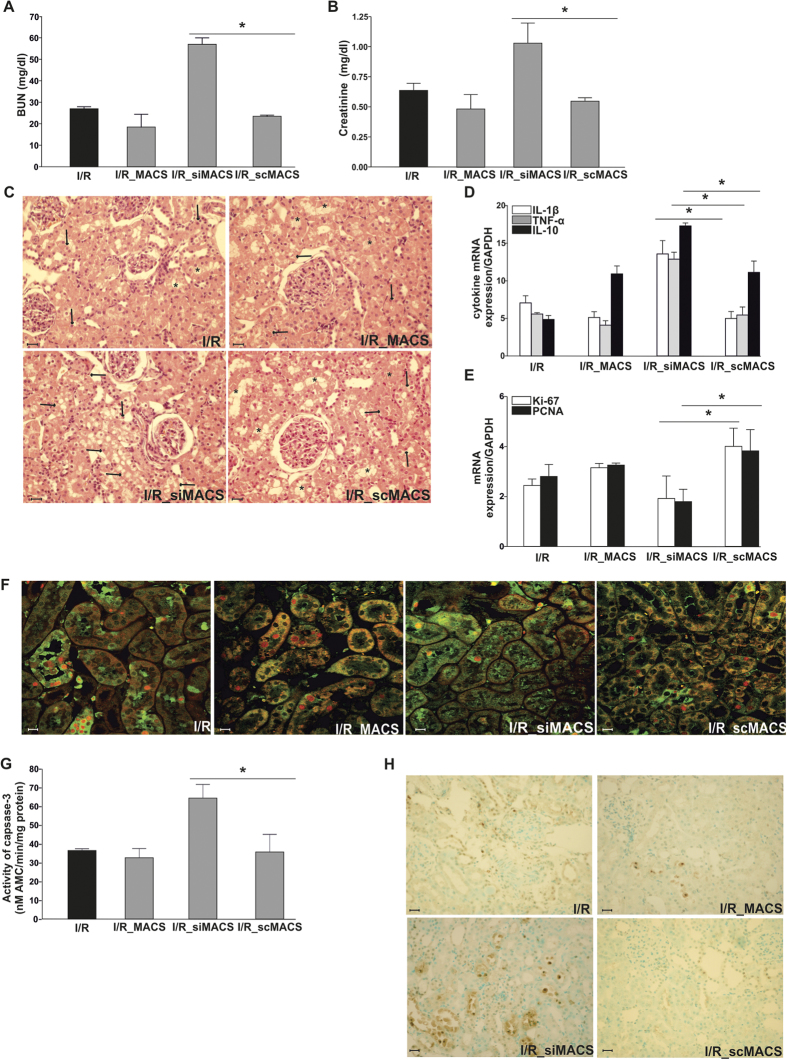
Lcn-2 knockdown macrophages promote IRI in Brown Norway rats. Animals were subjected to 45 min. of bilateral ischemia or were sham-operated and sacrificed at 24 h of reperfusion. The effect on (**A**) blood urea nitrogen (BUN) and (**B**) creatinine in plasma of adoptive transfer of either scrambeled (sc)RNA- or siLcn-2-treated macrophages was assessed. Macrophage infusion was performed for each condition at 1 h of reperfusion. (**C**) Conventional histological analysis of H&E-stained kidney sections (original magnification x40) of the cortico-medullar region confirmed BUN and creatinine data showing an enhanced injury profile in the siLcn-2-treated macrophage group. Representative images are shown and compared with I/R_scMACS group. Arrows indicate epithelial cell balloonization and tubular dilatation and asterisks show epithelial necrosis, detachment, and oedema (**D**) TNF-α, IL-1β, and IL-10 kidney expression profiles as representative inflammatory milieu of the different study groups was measured by mRNA expression via qRT-PCR from whole tissue homogenates. Results show an increase of pro-inflammatory cytokines due the adoptive transfer of Lcn-2 knockdown macrophages as compared to scRNA-treated macrophage infusion. The effect of (**E**) Ki-67 and PCNA mRNA expression (tested via qRT-PCR in total renal tissue homogenates) and (**F**) immunostaining of stathmin (green) and PCNA (red) expression (original magnification x400), in renal tissue of adoptive transfer of either scRNA- or siLcn-2-treated macrophages was assessed. Results show a significant decrease of regenerative/proliferative parameters in the Lcn-2 knockdown group compared to the scRNA macrophage group. The induction of apoptosis was assessed by measuring (**G**) activity of caspase-3 and by means of (**H**) the detection of TUNEL positive cells in whole kidney. Results show enhanced apoptosis in Lcn-2 knockdown macrophage group as compared to scRNA-treated macrophage infusion. Real-Time PCR data was normalized to the housekeeping gene GAPDH and expressed as arbitrary units of relative expression. Data is represented as means ± S.E.M. n = 5; *p < 0.05 vs IR_scMACS. bar = 50 μm.

BUN (Fig. 4A) and creatinine (Fig. 4B) indicated an essential role for Lcn-2 in macrophage-based therapy. siLcn-2-treated macrophages increased both injury markers as compared to the transfer of either non-transfected or scRNA-transfected control macrophages. H&E staining (Fig. 4C) and histological analysis (Table 1) further corroborated these results. Transfection efficiency using three different siRNA clones shows a significant decrease in Lcn-2 expression, both at mRNA and protein level (Figure S2A and S2B). LPS-treated macrophages were used as a positive control and Lcn-2 expression was determined 48 h post transfection. We also measured IL-10, Mannose Receptor, TNF-α, and IL-1β production in siRNA transfected macrophages. Results show a significant down-regulation of anti-inflammatory mediators (Figure S2C, S2D, and S2G) and an up-regulation of pro-inflammatory mediators in siLcn-2-macrophages. This was even more pronounced when stimulated with LPS (Figure S2E, S2F, and S2H).

The cytokine profile (Fig. 4D) of whole renal tissue also showed up-regulation of inflammation in Lcn-2-knockdown macrophage-treated animals as compared to the infusion of either non-treated or scRNA-treated control macrophages.

The pattern of Ki-67 and PCNA mRNA expression (Fig. 4E) indicates that a knockdown of Lcn-2 in infused macrophages impairs renal repair. Further, immunostaining for PCNA and stathmin corroborated these data (Fig. 4F). Quantification of PCNA- and stathmin-positive cells by counting at least five different fields further corroborated these results (Table 2).

Considering the pronounced tissue injury after adoptive transfer of Lcn-2-knockdown macrophages, we next evaluated the activity of caspase-3 in whole renal tissue homogenates (Fig. 4G). Caspase-3 activity was significantly induced after infusion of Lcn-2-knockdown macrophages, whereas neither non-transfected nor scRNA-treated control macrophages increased apoptosis following caspase-3 activity as compared to I/R group. Figure 4H shows determination of apoptosis *in situ* via immunohistochemical staining (TUNEL assay). In line with the caspase-3 activity profile, the adoptive transfer of Lcn-2-knockdown macrophages increased the number of TUNEL-positive apoptotic cells.

## Discussion

The aim of the study was to define if endogenous resistance to ischemic injury could be influenced by the nature of infiltrating macrophages. We point out that the expression of Lcn-2 from infiltrated macrophages is determinant to define IRI susceptibility. Adoptive transfer experiments of *ex vivo* modulated macrophages indicate a key role for Lcn-2 in cytoprotective mechanisms upon renal ischemia.

Acute renal failure, most commonly induced by ischemia/reperfusion injury, is still a vast clinical problem, and currently there are no specific therapies available to prevent injury or improve recovery. Mechanisms of protection and repair from renal injury are closely interlinked with pathways of ischemia/reperfusion injury. Recently, different approaches of studying inherited resistance to injury from ischemia/reperfusion in different strains from the same species have gained insight into pathophysiologic and putative cytoprotective mechanisms. Baker *et al.* showed that hearts from Brown Norway rats were more resistant to ischemic cardiac injury than hearts from other strains^20^ indicating a genetic basis of intrinsic resistance to injury. In line with Basile *et al.*^21^, we found strain-specific, intrinsic differences in levels of injury after 45 minutes of bilateral ischemia. Sprague Dawley rats presented a higher degree of injury markers blood urea nitrogen (BUN) and creatinine in plasma, whereas Brown Norway rats were essentially protected from injury and showed preserved kidney integrity. Despite recent advances, the mechanisms implicated in inherited protection in Brown Norway rats in response to ischemia/reperfusion injury are still unknown. Previously, we conducted a comparative genomic study in Brown Norway rats versus Sprague Dawley rats^7^. By selective manipulation of the Wnt pathway, we showed the implication of the Wnt target gene osteopontin in the maintenance of renal tissue integrity by down-regulation of mitochondrial cytochrome c release, and thus protection from apoptosis. Although it seems reasonable that differences in the genetic signature would offer resistance to different pathological situations, the mechanisms related to the upregulation of those genes and subsequent proteins that could offer such protection is still not well defined. However, we could speculate that Lcn-2 upregulation of macrophages in BN rats is linked directly or indirectly to the manipulation of the Wnt pathway, as seen also in other models of proliferation and differentiation^22^.

Macrophage infiltration plays a key role in initiation and orchestration of acute inflammatory responses in renal injury and may be critical to the outcome of ischemic renal injury. Published work of our group clearly indicates that macrophages are involved in renal regeneration after ischemia/reperfusion injury depending on the inflammatory milieu^11^. Under the appropriate stimulus, macrophages may change their phenotype from a classically activated pro-inflammatory to an alternative, anti-inflammatory one, thus helping to preserve cell survival and proliferation, finally leading to tissue healing. Activation/deactivation of macrophages determines the balance of tissue healing versus tissue injury. Interestingly, when macrophages were genetically modified *ex vivo* in order to express a predetermined phenotype towards anti-inflammation and were then reinjected, a protective role was clearly shown. Thus, control of the infiltrating macrophage phenotype, and therefore inflammatory outcome, may permit augmentation of regenerative mechanisms in damaged tubular epithelia, since the inflammatory environment modulates repair in tubular epithelial cells.

Although it seems clear that alternative phenotype activation is the suitable macrophage status for the use of macrophages in cellular therapy, there are not many attempts to establish new genes or candidate proteins to be modified in the macrophage for these purposes. Taken into account the possible intrinsic resistance of Brown Norway rats and the crucial role of macrophages in injury and repair, we first examined whether the difference could be explained by the efficacy in macrophage recruitment, but as shown in Fig. 2, there are no differences in the ability of recruitment to the injury site between the two strains. Furthermore, the BMDM isolation experiments revealed that the cells had the same degree of macrophage maturity, but respond differently towards the stress insult, as could be observed in the different production of inflammatory and anti-inflammatory proteins upon hypoxia and different Lcn-2 production. Thus, although the ability for macrophage recruitment after I/R injury could be very similar between the two strains, the behaviour of the recruited macrophages is different and our hypothesis is that Lcn-2 is a determinant in this setting.

Some authors reported different for the role of Lcn-2 in determining the macrophage phenotype. Recently, Jang *et al.* showed that Lcn-2 enhances brain inflammation due to the induction of M1-polarization of brain microglial cells^23^. Recent findings suggest that Lcn-2 acts as a regulator of macrophage polarization in obesity-associated inflammation, whereby macrophages from Lcn-2^−/−^ mice displayed less pro-inflammatory and increased anti-inflammatory markers^24^. Furthermore, it was described that Lcn-2 is mainly up-regulated in deactivated macrophages^25^. It might be speculated that the role of Lcn-2 in such specialized tissues such as the brain might differ substantially from other organs and tissue-context specific modes of action might be responsible for the observed dual role of Lcn-2 in the macrophage polarization process. But what is clear is that the inflammatory milieu is determinant to both, macrophage polarization and the effects of Lcn-2 in macrophage cells.

Our previous data support the role of Lcn-2 in determining macrophage phenotype polarization towards the anti-inflammatory M2 phenotype^9^,^26^. IL-10 stimulated primary human macrophages up-regulated Lcn-2 as being part of their pro-tumoral M2-like signature^26^, whereas the knockdown of Lcn-2 clearly impaired anti-inflammatory cytokine production in macrophages upon apoptotic cell challenge, thus impeding the beneficial role of Lcn-2 for renal ischemia/reperfusion injury outcome^9^.

In our experimental, the cytokine profile from the two examined strains was profoundly different, both under resting and under hypoxia-stimulated conditions. In this line, a reasonable question might arise regarding the potential role of Lcn-2 in resident renal mononuclear phagocytes, which are exposed to the ischemic insult and the lack of oxygen, versus the infiltrating monocytes/macrophages that home to the kidney during the reperfusion phase, therefore having access to oxygen. But the question is reduced if we consider that finally both types of macrophages are exposed to the same inflammatory environment induced by the reperfusion period. In both cases, the effect of Lcn-2 might be similar due to the same milieu, which is in fact crucial of the reparative role of Lcn-2, as seen previously in our group^11^,^19^.

We could further show that the resolution of inflammation *in vivo* was more efficient in Brown Norway than in Sprague Dawley rats. These findings are corroborated by the study of Sáenz-Morales *et al.*^8^ where they clearly observe faster resolution of inflammation in Brown Norway rats with decreased levels of pro-inflammatory cytokines MCP-1, IL-1β, and TNF-α. It is therefore feasible to think that the difference between the sensitivity and resistance to ischemia could be due to intrinsic differences of macrophages.

In this line, we observed higher levels of the pro-proliferative and pro-regenerative mediator Lcn-2 in macrophages isolated from Brown Norway rats. We previously described a role for macrophage-derived Lcn-2 in promoting kidney repair and proliferation of renal epithelial cells^9^,^19^. Lcn-2 was also described by others to modulate renal repair and, therefore, kidney integrity. In the current study, we found that renal regeneration after ischemia/reperfusion injury is promoted by macrophage-generated Lcn-2 in IRI-sensitive Sprague Dawley rats, while Lcn-2 gene knockdown in macrophages provoked a significant loss of cytoprotection in IRI-resistant Brown Norway rats. More concretely, *ex vivo* genetically modified macrophages overexpressing Lcn-2 were capable of modulating both injury and inflammation outcome in IRI-sensitive ischemic kidneys. Renal injury markers BUN and creatinine were significantly decreased upon adoptive transfer of these macrophages and the expression of pro-inflammatory mediators was attenuated. On the contrary, administration of Lcn-2-silenced macrophages induced tissue injury and promoted higher levels of both blood urea nitrogen and creatinine in plasma in IRI-resistant ischemic kidneys. Besides, apoptosis induction after knockdown-macrophage transfer was significantly increased and was in line with the observed histological kidney damage. These results reinforce the hypothesis that Lcn-2 may play a pivotal role in macrophage-dependent reparative mechanisms during renal ischemia/reperfusion injury. Another important point was that the inflammatory milieu of the tissue after the adoptive transfer of *ex vivo* manipulated macrophages. We found a microenvironment associated to the anti-inflammatory status of Lcn-2 overexpressing macrophages. Interestingly, although the injected “programmed” anti-inflammatory macrophages comprise only a small number of total infiltrated macrophages, they still possess the ability to reduce injury and probably, to reorient the phenotype of other infiltrated immune cells, since the tissue environment remains anti-inflammatory after the cell therapy protocol. The data in supplementary figures S1 and S2 also depict the ability of macrophages to be “re-programmed” in order to maintain the anti-inflammatory status, although they undergo a pro-inflammatory treatment with LPS.

In conclusion, we hypothesize that mechanisms associated to ischemic resistance in Brown Norway rats may be regulated by the intrinsic expression of Lcn-2 from infiltrating macrophages. Concretely, by the use of adoptive transfer studies of *ex vivo* genetically modified macrophages, overexpressing or silenced for Lcn-2, we delineated a clear picture of the importance of macrophage-Lcn-2 upon renal ischemia/reperfusion injury. Our study further provides evidence that macrophage-derived Lcn-2 not only has an anti-inflammatory effect on the ischemic kidney, but also promotes the proliferation and regeneration of tubular epithelial cells. These findings may give further insight into the pathophysiological processes responsible not only for organ dysfunction, but also cytoprotective mechanisms secondary to ischemia/reperfusion injury. Understanding the mechanisms that regulate genes, such as Lcn-2, related to cytoprotective pathways could offer new perspectives for more efficient therapy of renal disease. Additionally, the use and therapeutic application of anti-inflammatory and reparative macrophages provides a more physiological gene delivery tool. Nevertheless, further research is needed to define the diverse biological effects of Lcn-2 within the kidney. Regarding its role in kidney regeneration, we are currently scrutinizing on the growth promoting effects of Lcn-2 from macrophages, since our study particularly points to the importance of macrophages in producing renal regeneration stimulating molecules like Lcn-2.

## Materials and Methods

### Animal Model and Ethic Statement

We used male Brown Norway rats (225–250 g) and male Sprague Dawley rats (225–250 g) (Charles River, France) for comparative studies on resistance to ischemia/reperfusion injury. All procedures were approved and further conducted under the supervision of our institution’s Research Commission and in accordance of the approved European Union guidelines for the handling and care of laboratory animals.

### Ischemia/Reperfusion injury (IRI)

Animals were anesthetized with isoflurane and placed in a supine position with body temperature maintained at 37 °C. After laparatomy and dissection of both renal pedicles, bilateral ischemia was induced by occluding the renal pedicles with microvascular clamps for 45 minutes. Completeness of ischemia was verified by the obscuring of the kidneys, indicating the blockade of blood flow. To minimize dehydration of the exposed tissues, the abdominal area was covered with saline-soaked gauze at 37 °C plus a plastic cover. Blood flow to the kidneys was reestablished by removal of the clamps (reperfusion), with visual verification of blood return and blanching of kidneys. Animals subjected to sham operation were used as controls. During the procedure, animals were well hydrated and body temperature was maintained around 37 °C. During the reperfusion period, animals were kept under observation by the veterinarian.

At the end of the reperfusion period, animals were killed, blood was collected, and kidneys were harvested and snap frozen at −80 °C or fixed in paraformaldehyde.

### Culture and adoptive transfer of bone marrow-derived macrophages

Rat bone marrow-derived cells were isolated by aspiration of femur and resuspended in DMEM 1:1 F-12 with high glucose, supplemented with 10% heat-inactivated fetal bovine serum, 10 ng/ml Granulocyte-macrophage colony-stimulating factor (GM-CSF), 100 U/ml penicillin and 100 μg/ml streptomycin and were kept in non-adherent culture flasks to mature for 7 days. Macrophages were subsequently isolated by adherence. Cells were kept in a humidified atmosphere of 5% CO_2_ in air at 37 °C.

For *in vitro* experiments, both cells and supernatant were collected for subsequent cytokine and Lcn-2 detection.

For *in vivo* experiments, cells from Brown Norway rats were either treated with siRNA to knockdown Lcn-2 in macrophages (**I/R + siMACS**), transfection vehicle (**I/R + MACS**), or a randomly chosen control siRNA (**I/R + scMACS**) 24 hours before injection. For overexpression experiments, matured bone marrow-derived macrophages were transduced with either adenoviral vector AdLcn-2 (**I/R_Lcn-2**) or the control vector Adβ-gal (**I/R_β-gal**). Transduction efficiency was routinely checked by qRT-PCR and ELISA.

Macrophages were smoothly transferred to a culture tube, and maintained in PBS until injection into the animal. We injected resting bone marrow-derived macrophages (BMDM) (10 × 10^6^ cells per rat, intravenously by direct puncture in the inferior cava) 1 hour after reperfusion induction.

### Hypoxia/Reoxygenation experiments

Cells were transferred at a density of 1 × 10^6^ cells to 60 mm Petri dishes and were subjected to hypoxic conditions (1% O_2_) (**H/R**) or to normoxic conditions (20, 7% O_2_) (**C**) for 16 hours. During the reoxygenation period of 4 hours, cells were kept in a humidified atmosphere of 5% CO_2_ in air at 37 °C.

Both the cell pellet and the supernatant were collected for further cytokine and Lcn-2 determination by PCR and ELISA.

### Adenoviral vectors and dose-response assay

Adenoviral vectors were applied in order to transduce macrophages *ex vivo*.

Adenoviral vectors for β-galactosidase (Adβ-gal) were kindly provided by Dr David Kluth and Dr Jeremy Hughes (MRC Centre for Inflammation Research, The Queen’s Medical Research Institute, Edinburgh, UK). The adenoviral vector carrying cDNA encoding recombinant Lcn-2 was elaborated, amplified, and purified by ViraQuest, Inc (North Liberty, IA, USA).

For dose-response assay, cells were collected, counted and aliquoted to obtain 1 × 10^6^ cells, transduced with lipofectamine, and the different indicated adenoviral vectors at different doses in order to determine the optimum dose which yields the most effective transduction, but at the same time has a reasonable balance between cytopathic effects of the virus and its efficiency. Cells were incubated for 48 hours at 37 °C in a CO_2_ incubator. The determined optimum multiplicity of infection (MOI100) was further used in the experiments.

### Flow cytometry for macrophage maturity

Matured bone marrow-derived macrophages from both strains were seeded at a density of 1 × 10^6^ cells. For flow cytometry analysis, macrophages were scraped out, centrifuged at 800 × g for 5 minutes and subsequently blocked with 10% mouse serum (Sigma, Madrid, Spain) for 30 minutes at 4 °C. CD11b-positive macrophages were detected by incubating cells with a FITC-conjugated monoclonal mouse anti-rat CD11b IgG antibody (MCA275FT; Serotec, Madrid, Spain) for 30 minutes at 4 °C. Specific labelling was compared with nonspecific staining using a FITC-labelled isotype-matched control antibody (eBioscience, Madrid, Spain). Labelled macrophage preparations were analyzed on a FACSCalibur using CellQuest software (BD Biosciences).

### Immunofluorescent staining for ED1 on matured bone marrow-derived macrophages

Matured bone marrow-derived macrophages from both strains were seeded at a density of 1 × 10^6^ cells on a coverslip. Cells were then fixed in 3.7% paraformaldehyde for 10 minutes, washed with PBS, permeabilized with acetone for 5 minutes on ice, and blocked with goat serum for one hour at room temperature. Cells were washed twice with PBS and incubated with monoclonal mouse anti-rat CD68 (ED1) (MCA341R; Serotec, Madrid, Spain) for 1 hour at room temperature. After washing with PBS, slides were incubated with secondary fluorescent antibody (1:1000) (Alexa fluor 546 Goat Anti-mouse, highly cross absorbed, Molecular Probes, USA) for 30 minutes. The preparation was mounted with mowiol (Calbiochem, La Jolla, CA) and cells were viewed using a Leica TCS NT laser microscope (Leica Microsystems, Wetzlar, Germany), equipped with a PT APO objective (60 ×).

### siRNA (Small Interfering RNA) Gene Silencing

The siRNA sequence used for targeted silencing of LCN-2 gene was synthesized using the BLOCK-iT RNAi Designer (Invitrogen, Barcelona, Spain). The *LCN-2* siRNA oligonucleotides selected were (sense strand given): siLCN-2_1: 5′-GCC CAG GAC TCA ACT CAG AAC TTG A-3′; siLCN-2_2: 5′-GCA CCA TCT ATG AGC TAC AAG AGA A-3′: siLCN-2_3: 5′-CAA GAG AAC AAT AGC TAC AAT GTC A-3′. A siRNA targeted to no known gene (Stealth RNAi negative control, Invitrogen, Barcelona, Spain) was used as a negative control (scRNA). Additionally, effective siRNA delivery was verified by fluorescent staining of transfected cells using the siRNA Reporter Block-it system (Invitrogen). The synthetic double-stranded siRNA oligonucleotides were delivered to macrophages using Lipofectamine 2000 according to manufacturer’s recommended protocol (Invitrogen). *Lcn-2* gene expression was measured by Real-Time RT-PCR at 24 and 48 hours post transfection, and also by assessing the amount of Lcn-2 protein secreted into the medium at these timepoints.

### Renal injury markers

Blood urea nitrogen (BUN) and creatinine were analysed in plasma using an ADVIA 2400 (Siemens Medical Diagnostics) multichannel analyzer at the Hospital Clínic, Barcelona.

### Caspase 3 activity

Caspase-3-like activity was determined by measuring proteolytic cleavage of the specific substrate *N*-acetyl-Asp-Glu-Val-Asp-7-amino-4-methylcoumarin (DEVD-AMC; Biomol, Plymouth Meeting, PA). Renal tissue was homogenated and sonicated in assay buffer (50 mM HEPES, 10% sucrose, 0.1% CHAPS, 5 mM GSSG, 5 mM DTT). We used 250 μg of protein of each sample and 12 μM DEVD-AMC to perform the assay. The AMC released was quantified for 2 hours at 37 °C by fluorospectrophotometry using 380 nm excitation and measurement of 450 nm emissions.

### Protein concentration

*T*otal protein concentration in homogenates was determined using a commercial kit from Bio-Rad (Munich, Germany).

### TUNEL staining

TUNEL assay in tissue sections were performed using the DNA fragmentation detection Colorimetric-TdT Enzyme Kit (Calbiochem, La Jolla, CA) and according to the manufacturer’s instructions. Sections were counterstained with methyl green stain, dehydrated through a graded series of alcohol, and mounted in DPX for microscopy analysis.

### Immunostaining of kidney sections

Kidneys were fixed in 4% paraformaldehyde, embedded in paraffin and cut in sections of 4 μm. Paraformaldehyde-fixed paraffin-embedded sections were washed in PBS and were blocked for 1 h using goat serum in PBS. Slides were subsequently incubated overnight at 4 °C with monoclonal antibody, macrophage marker (3H2617) (Santa Cruz Biotechnology, Barcelona, Spain). Tissue sections were then rinsed in PBS and incubated with secondary goat anti-mouse IgG antibody conjugated with Alexa Fluor 568 (Molecular Probes, Barcelona, Spain) for 2 hours at room temperature. Sections were mounted with mowiol (Calbiochem, Madrid, Spain) and viewed using a Leica TCS NT laser microscope (Leica Microsystems, Wetzlar, Germany). Macrophages were quantified by counting the number of positive cells per 100 cells counted in an average of five high-power fields (x40) in each section. Previous authors have defined stathmin-positive cells as a marker of dedifferentiated, mitotically active in epithelial cells and that could contribute to tubular regeneration^27^. Kidney sections were unmasked in sodium citrate buffer and blocked for 1 h for subsequent immunofluorescent stainings. Staining of stathmin and PCNA were prepared as previously described^10^. Slides were incubated with anti-stathmin (Calbiochem) and anti-PCNA antibody (Santa Cruz), followed by incubation with secondary antibodies (Molecular Probes) for 2 h. To detect CD206 expression, slides were incubated with a FITC-labelled anti-CD206 antibody (BD Bioscience, Heidelberg, Germany) overnight at 4 °C. For iNOS detection, kidney sections were unmasked as described above and stained by applying a ployclonal iNOS antibody (Enzo Life Science, Lörrach, Germany) and by using the Catalyzed Signal Amplification System (DAKO, Hamburg, Germany) based on the streptavidin–biotin peroxidase reaction, according to the instructions provided by the manufacturer.

### Real-Time RT-PCR

Total kidney RNA was isolated from homogenized tissue with TRIzol Reagent (Invitrogen) according to manufacturer’s instructions. Total RNA from cells was isolated using the RNeasy mini kit following the manufacturer’s protocol (Qiagen, Barcelona, Spain). RNA concentrations were calculated from A_260_ determinations. The purity and quality of extracted RNA were evaluated using the RNA 6000 LabChip and Agilent 2100 Bioanalyzer (Agilent Technologies, Palo Alto, CA, USA). Quantitative RT-PCRs were performed in a Bio-Rad iCycler iQ Real-Time-PCR detection system using SYBR Green RT-PCR detection Kit (Bio-Rad, Madrid, Spain) according to manufacturer’s instructions. Proliferation data were obtained by *Ki-67* expression: forward, AGA CGT GAC TGG TTC CCA AC; reverse, ACT GCT TCC CGA GAA CTG AA, and by *PCNA* determination: forward, AGG ACG GGG TGA AGT TTT CT; reverse, CAG TGG AGT GGC TTT TGT GA.

*Lcn-2* data were collected using the following primer set: forward, CAA GTG GCC GAC ACT GAC TA; reverse, GGT GGG AAC AGA GAA AAC GA. *TNF-α* expression was obtained by using the following primer set: forward, AAC TCC CAG AAA AGC AAG CA; reverse, CGA GCA GGA ATG AGA AGA GG and the following primer set for Mannose receptor expression: forward, GCA GGT GGT TTA TGG GAT GT; reverse, GGG TTC AGG AGT TGT TGT GG (all primers were purchased from Invitrogen, Barcelona, Spain).

Cytokine data for *IL-1β* and *IL-10* were collected using validated primers provided by Qiagen (Barcelona, Spain).

Results were normalized to glyceraldehyde-3-phosphate dehydrogenase (*GAPDH*) as internal control for stable expression (housekeeping gene): forward, CCC CCA ATG TAT CCG TTG TG; reverse, TAG CCC AGG ATG ATG CCC TTT AGT.

### Protein detection by ELISA

Supernatants were collected and clarified by centrifugation. 50 μl of each sample were applied to an ELISA 96-well-plate previously covered with catch Anti-mouse Lipocalin-2/Lcn-2 monoclonal antibody (Vitro, Madrid, Spain) and blocked for 1 hour. After sample incubation, detection biotinylated anti-mouse Lipocalin-2/Lcn-2 antibody (Vitro, Madrid, Spain) was added. Afterwards, HRP-conjugated avidin (Invitrogen, Barcelona, Spain) was incubated for 1 hour. Finally, color reagent (OPD tablets, Dako, Glostrup, Denmark) was added and color development was assessed by a microplate reader.

For cytokine levels of TNF-α and IL-10, supernatants were processed according to manufacturer’s kit instructions (Endogen, Pierce Biotechnology, Barcelona, Spain).

### Statistical Analyses

Data were analyzed using analysis of variance (ANOVA) followed by Newman-Keuls test analyses. Data are presented as the means ± S.E.M with *p* values less than 0.05 considered significant.

## Additional Information

**How to cite this article**: Jung, M. *et al.* Macrophage-derived Lipocalin-2 contributes to ischemic resistance mechanisms by protecting from renal injury. *Sci. Rep.* **6**, 21950; doi: 10.1038/srep21950 (2016).

## Supplementary Material

Supplementary Information

## Figures and Tables

**Table 1:** Histopathological analysis of renal injury in Brown Norway versus Sprague Dawley rats.

Experimental group	Epithelial necrosis	Epithelial balloonization	Tubular dilatation	Detachment	Oedema	Total score
Brown Norway rats						
Sham	0.3+/−0.1	0.4+/−0.01	0	0.2+/−0.03	0	0.9+/−0.14
I/R_24 h	2.0+/−0.1	1.3+/−0.04	0.8+/−0.1	1.9+/−0.4	0.1+/−0.03	6.1+/−0.67^*^
I/R_MACS	1.2+/−0.4	0.9+/−0.08	0.4+/−0.05	0.9+/−0.06	0.6+/−0.06	4.0+/−0.65
I/R_siMACS	2.4+/−0.2	1.5+/−0.08	1.0+/−0.05	2.5+/−0.05	0.6+/−0.06	8.4+/−0.44^+^
I/R_scMACS	1.3+/−0.3	0.8+/−0.1	0.5+/−0.04	0.8+/−0.04	0.6+/−0.06	4.0+/−0.54
Sprague Dawley rats						
Sham	0.6+/−0.03	0.4+/−0.03	0.2+/−0.01	0.5+/−0.03	0	1.7+/−0.1
I/R_24 h	2.9+/−0.1	1.5+/−0.05	1.9+/−0.02	2.5+/−0.2	0.4+/−0.03	9.2+/−0.6
I/R_MACS	0.8+/−0.6	0.4+/−0.08	0.7+/−0.05	1.9+/−0.06	0.2+/−0.06	4.0+/−0.06
I/R_Lcn-2	0.7+/−0.6	0.3+/−0.08	0	0.3+/−0.06^+^	0	1.3+/−0.74^§^
I/R_β-gal	1.0+/−0.06	0.6+/−0.08	0.8+/−0.02	1.3+/−0.06	0.4+/−0.05	4.1+/−0.27

Histological analysis indicates that BN rats, as compared to SD rats, preserve significantly higher tissue integrity after ischemia/reperfusion injury. Less cellular infiltration, tubular cell balloonization, and severe necrosis could be observed. Transfer of Lcn-2 knockdown macrophages to BN animals promoted renal injury, whereas transfer of Lcn-2 over-expressing macrophages to SD rats significantly enhanced protection against IRI. Data are represented as means ± S.E.M. n = 5; *p < 0.05 BN I/R_24 h vs. SD I/R_24 h, ^+^p < 0.05 BN I/R_siMACS vs. BN I/R_scMACS, ^§^p < 0.05 SD I/R_Lcn-2 vs. SD I/R_β-gal.

**Table 2:** Analysis of renal regeneration markers in Brown Norway versus Sprague Dawley rats.

Experimental group	PCNA-positive	Stathmin positive
Brown Norway rats		
Sham	1+/−0.5	2+/−0.2
I/R_24 h	7+/−0.6^*^	9+/−0.8^*^
I/R_MACS	6+/−0.9	8+/−0.7
I/R_siMACS	3+/−0.5^+^	2+/−0.1^+^
I/R_scMACS	8+/−0.9	7+/−0.7
Sprague Dawley rats		
Sham	1+/−0.3	1+/−0.1
I/R_24 h	4+/−0.2	5+/−0.2
I/R_MACS	8+/−0.4	7+/−0.5
I/R_Lcn-2	18+/−1.2^§^	15+/−1.5^§^
I/R_β-gal	7+/−0.9	5+/−0.6

The numbers depict averaged PCNA- and stathmin-positive cell counts per field. 5 fields were counted per section. Results indicated that BN rats, as compared to SD rats, represent significantly higher renal cell proliferation after ischemia/reperfusion injury. Transfer of Lcn-2 knockdown macrophages to BN animals induced a loss of regeneration markers at 24 h of reperfusion, whereas transfer of Lcn-2 over-expressing macrophages to SD rats significantly promoted renal regeneration. Data are represented as means ± S.E.M. n = 5; *p < 0.05 BN I/R_24 h vs. SD I/R_24 h, ^+^p < 0.05 BN I/R_siMACS vs. BN I/R_scMACS, ^§^p < 0.05 SD I/R_Lcn-2 vs. SD I/R_β-gal.
