# Supplementary material for: Macrophage-derived Lipocalin-2 contributes to ischemic resistance mechanisms by protecting from renal injury
Source: Sci Rep. 2016 Feb 25;6:21950. doi: 10.1038/srep21950 (PMC4766505; doi:10.1038/srep21950)
Supplement: Supplementary Information [file srep21950-s1.pdf]

SUPPLEMENTAL MATERIAL

Macrophage-derived Lipocalin-2 contributes to ischemic resistance mechanisms by protecting from renal injury

Michaela Jung, Bernhard Brüne, Georgina Hotter, Anna Sola*

GROUP	Viable cells (%)	Dead cells (%)
Normoxia	94.6 ±2.1	5.4 ±2.1
16h Hypoxia/ 4h Re-oxygenation	93.1 ±2.4	6.9 ±2.4
18h Hypoxia/ 4h Re-oxygenation	83.7 ±2.7	16.3 ±2.7
20h Hypoxia/ 4h Re-oxygenation	70.7 ±4.8*	29.3 ±2.3*
24h Hypoxia/ 4h Re-oxygenation	69.3 ±6.4*	30.68 ±2.9*

Supplementary table 1.- Cellular viability

The Alamar Blue Assay assesses cell viability of BMDM by measuring the integrity of the cell membrane. By counting the number of blue-coloured cells, a loss of cell viability and cell growth was indicated. Data are represented as means of the percentage of total cell number ± S.E.M; n=6, *p<0.05 *vs.* Normoxia

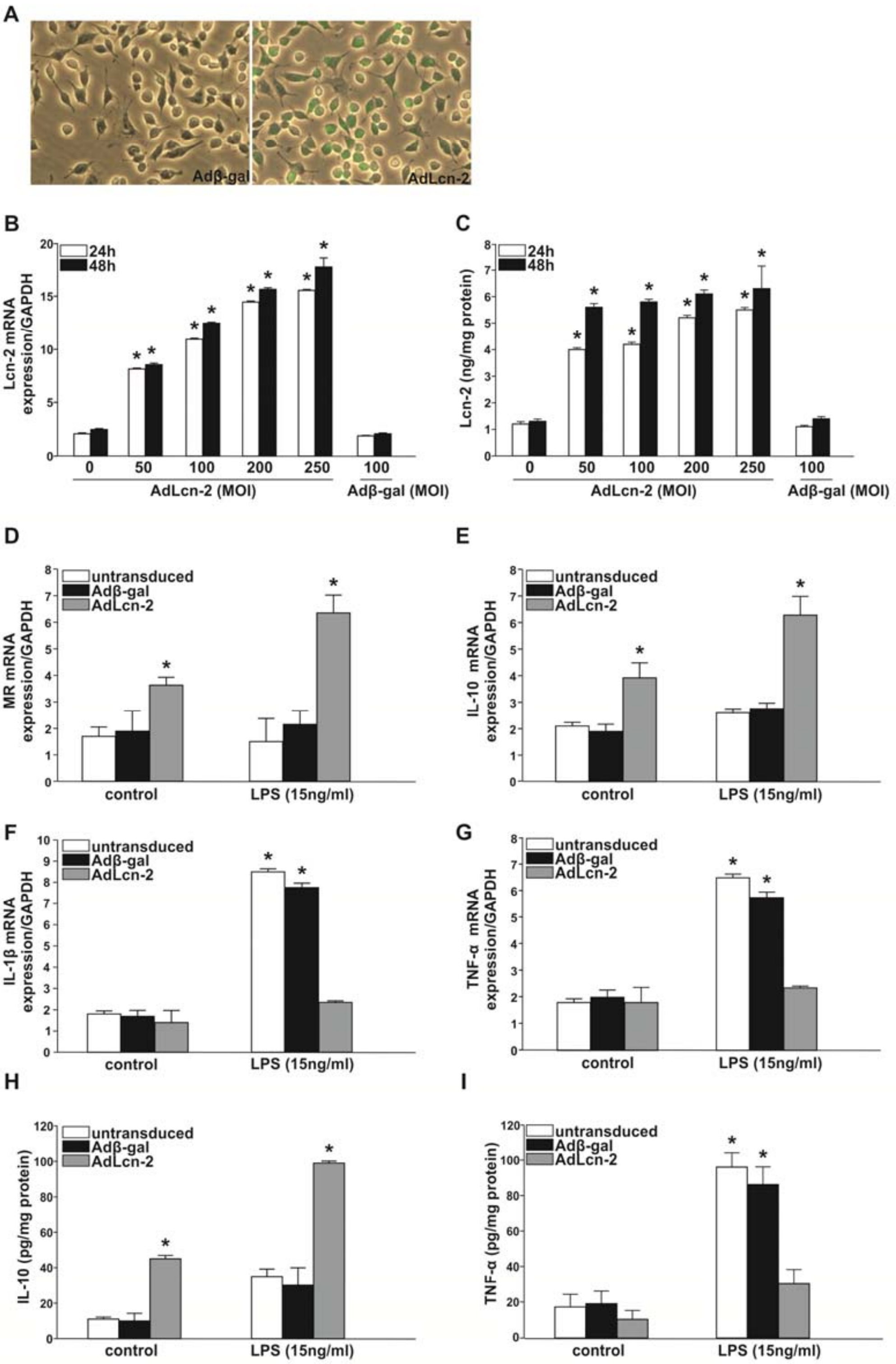

FIGURE S1.- Lcn-2-overexpressing macrophages from Sprague Dawley rats exhibit a predominant anti-inflammatory phenotype

(A) Transduction efficiency of BMDM was measured by transduction with a GFP-tagged adenoviral vector expressing Lcn-2 and viewed under a fluorescent microscope. AdLcn-2-transduced BMDM show a dose- and time-dependent increase in Lcn-2 expression, both on **(B)** mRNA level measured by qRT-PCR and on **(C)** protein level measured by ELISA. Transduced macrophages were either left untreated or stimulated with LPS (10ng/ml) for 24h to verify their inflammatory response. **(D)** Mannose receptor and **(E)** IL-10 expression as representative anti-inflammatory markers and **(F)** IL-1ß and **(G)** TNF-α expression as representative pro-inflammatory markers were measured by real-time qRT-PCR from transduced BMDM. Results show a clear up-regulation of anti-inflammatory mediators in Lcn-2 over-expressing macrophages, indicating a predominant anti-inflammatory macrophage phenotype, even when stimulated with the pro-inflammatory mediator LPS. mRNA expression data could be confirmed on protein level by measuring **(H)** IL-10 and **(I)** TNF-α by ELISA. Data are represented as means ± S.E.M. n=10; qRT-PCR data is represented as arbitrary units of relative gene expression normalized to the housekeeping gene GAPDH. *$p<0.05$ vs. MOI 0/Control.

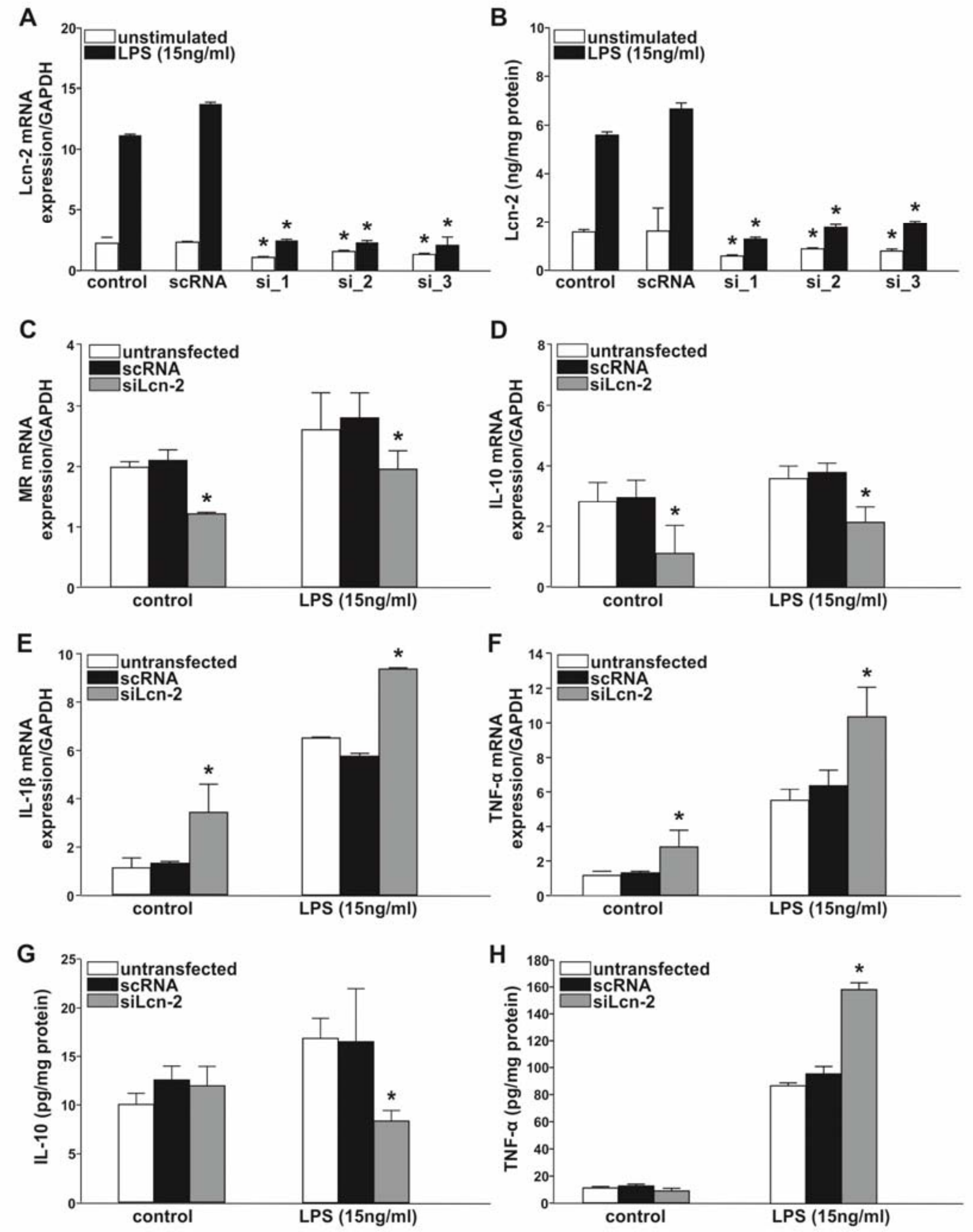

FIGURE S2.- Lcn-2-knockdown in Brown Norway macrophages promotes a predominant pro-inflammatory phenotype

Transfection efficiency of bone marrow-derived macrophages was measured both on **(A)** mRNA level by Real-Time qRT-PCR and on **(B)** protein level by ELISA and siLcn-2-treated macrophages were compared to scRNA-treated macrophages. Transfected macrophages were either left untreated or stimulated with LPS (10ng/ml) for 24h to verify their inflammatory response. **(C)** Mannose receptor and **(D)** IL-10 expression as representative anti-inflammatory markers and **(E)** IL-1ß and **(F)** TNF-α expression as representative pro-inflammatory markers were measured by qRT-PCR from transfected BMDM. Results show a clear up-regulation of pro-inflammatory mediators in Lcn-2 knockdown macrophages, indicating a predominant pro-inflammatory macrophage phenotype, even more pronounced after stimulation with LPS. Results from protein measurements using ELISA for **(G)** IL-10 and **(H)** TNF-α confirmed mRNA expression data. Data are represented as means ± S.E.M. n=10; qRT-PCR data is represented as arbitrary units of relative gene expression normalized to the housekeeping gene GAPDH. *$p<0.05$ vs. scRNA.

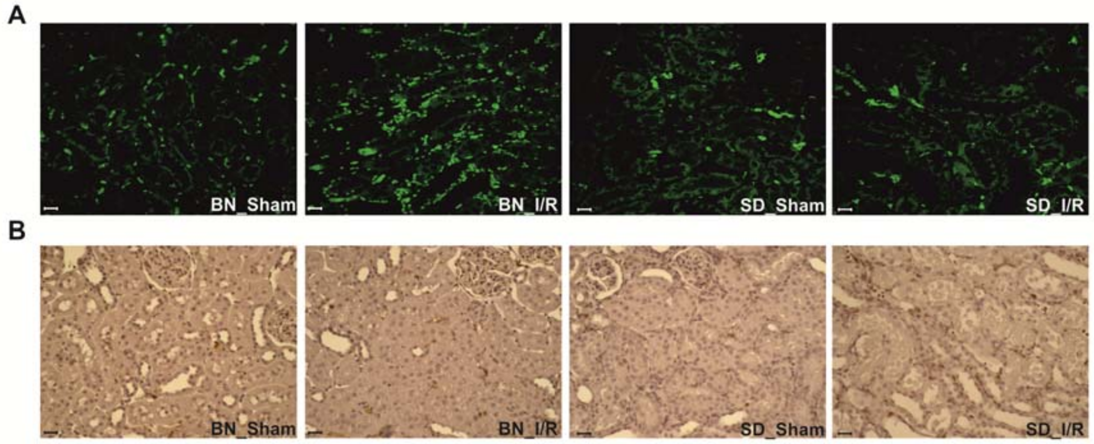

FIGURE S3.- Assessment of iNOS and CD206 expression in Brown Norway and Sprague Dawley rats

Animals were subjected to 45min. of bilateral ischemia or were sham-operated and sacrificed at 24h of reperfusion. **(A)** Immunostaining of CD206 (green) and **(B)** iNOS (brown) (original magnification x400) was assessed in renal tissue. Results show significantly more CD206 positive cells in Brown Norway rats after 24h of reperfusion, whereas a decrease of iNOS expression was detected compared to Sprague Dawley rats.
